# Snowmelt and laying date impact the parental care strategy of a high-Arctic shorebird

**DOI:** 10.1038/s41598-025-02318-y

**Published:** 2025-06-05

**Authors:** Léa Etchart, Nicolas Lecomte, François-Xavier Dechaume-Moncharmont, Johannes Lang, Jérôme Moreau, Thomas Pagnon, Niels Martin Schmidt, Benoit Sittler, Loïc Bollache, Olivier Gilg

**Affiliations:** 1 Université Marie et Louis Pasteur, CNRS, Chrono-Environnement (UMR 6249), 25000 Besançon, France; 2 Canada Research Chair in Polar and Boreal Ecology and Centre d’Études Nordiques, Université de Moncton, Moncton, NB Canada; 3 Universite Claude Bernard Lyon 1, CNRS, ENTPE, UMR 5023, Villeurbanne, France; 4 Working Group for Wildlife Research at the Clinic for Birds, Reptiles, Amphibians and Fish, Justus Liebig, University Giessen, 35392 Giessen, Germany; 5 Groupe de Recherche en Écologie Arctique, 21440 Francheville, France; 6 UMR 6282 CNRS, Université de Bourgogne, 6 Boulevard Gabriel, Dijon, France; 7 Centre d’Études Biologiques de Chizé, CNRS, 79360 Villiers en Bois, France; 8 Department of Ecoscience and Arctic Research Centre, Aarhus University, Roskilde, Denmark; 9 Chair for Nature Conservation and Landscape Ecology, University of Freiburg, Freiburg, Germany

**Keywords:** Biparental, Uniparental, Proximal factors, Sanderling, Greenland, Path analysis, Behavioural ecology, Population dynamics

## Abstract

Why parental care strategies can vary from uniparental to biparental care across taxa remains unclear, likely because various sets of ecological conditions are at-play. Here we tested ten possible hypotheses to decipher the direct and indirect impacts of critical factors likely to influence the parental care strategy during incubation in Sanderlings (*Calidris alba*), one of the few species that uses both types of care during that critical time of the breeding cycle. We examined three ecological factors (timing of local snowmelt, regional temperatures, and the North Atlantic Oscillation experienced just before breeding), one trophic factor (predation pressure), and two social factors (relative abundance of Sanderlings and their laying dates). Using long-term data from Greenland (2011–2023), path analyses revealed that laying date and snowmelt influence parental care strategies during incubation, with indirect climatic effects during migration and on breeding grounds. We observed a greater proportion of uniparental nests in years with delayed laying dates, and the reverse in years with delayed snowmelt. These findings underscore the complex interplay between environmental parameters and parental care strategies, offering insights into how these strategies are likely to respond to rapidly changing Arctic ecological conditions driven by climate change.

**Supplementary Information:**

The online version contains supplementary material available at 10.1038/s41598-025-02318-y.

## Introduction

Parental care, defined as any parental behaviour that enhances the offspring’s fitness, is an essential feature of animal breeding systems^1^. These parental traits are expected to evolve when the benefits of increased offspring survival outweigh the costs of providing care^2^. Parental care displays significant diversity across animal taxa from insects to mammals (e.g.,^3,4^), and spans a continuum from uniparental care by either males or females to strictly biparental care^1^. While the majority of birds exhibit biparental care (81%^5^), mammals predominantly display uniparental care^2^, while bony fishes and amphibians display a large diversity of care strategies, e.g.^6^.

Understanding parental care strategies and its determinants is crucial because in many species, reproductive success relies on the type of care, and the number of parents involved^1,2,7^. Comparative analyses and field studies investigated the evolution and determinants of parental care diversity. Ecological and trophic factors such as weather, predation, food and nest site availability, as well as social environment (density, sex-ratio, availability of partners) seem to influence the parental care strategy^6,8–13^. Harsh environment hypothesis states that under inclement conditions e.g., extremely dry, cold or hot weather, one parent could not provide sufficient care on its own^8,12^. On the other hand, biased sex ratio seem to decrease parental cooperation, due to greater re-mating opportunities for the scarcer sex^9^. However, few studies have examined the variability between individuals of the same population^14,15^. While for some species, parental care is fixed among individuals, others demonstrate variability between individuals. Zheng et al*.*^15^ described that social environment, i.e., breeding timing and re-mating opportunities impacted parental care strategies in Chinese penduline tits (*Remiz consobrinus)*. Nevertheless, the effect of ecological factors such as weather conditions, or trophic factors like predation or food availability, have never been tested on how parental care strategies are expressed, let alone tested together for potential cumulative effects. It appears fundamental to decipher the mechanisms and determinants of the parental care strategy at the inter-individual level to further understand how both uni- and biparental strategies can be maintained in a population if uniparental care is successful.

**Fig. 1:**
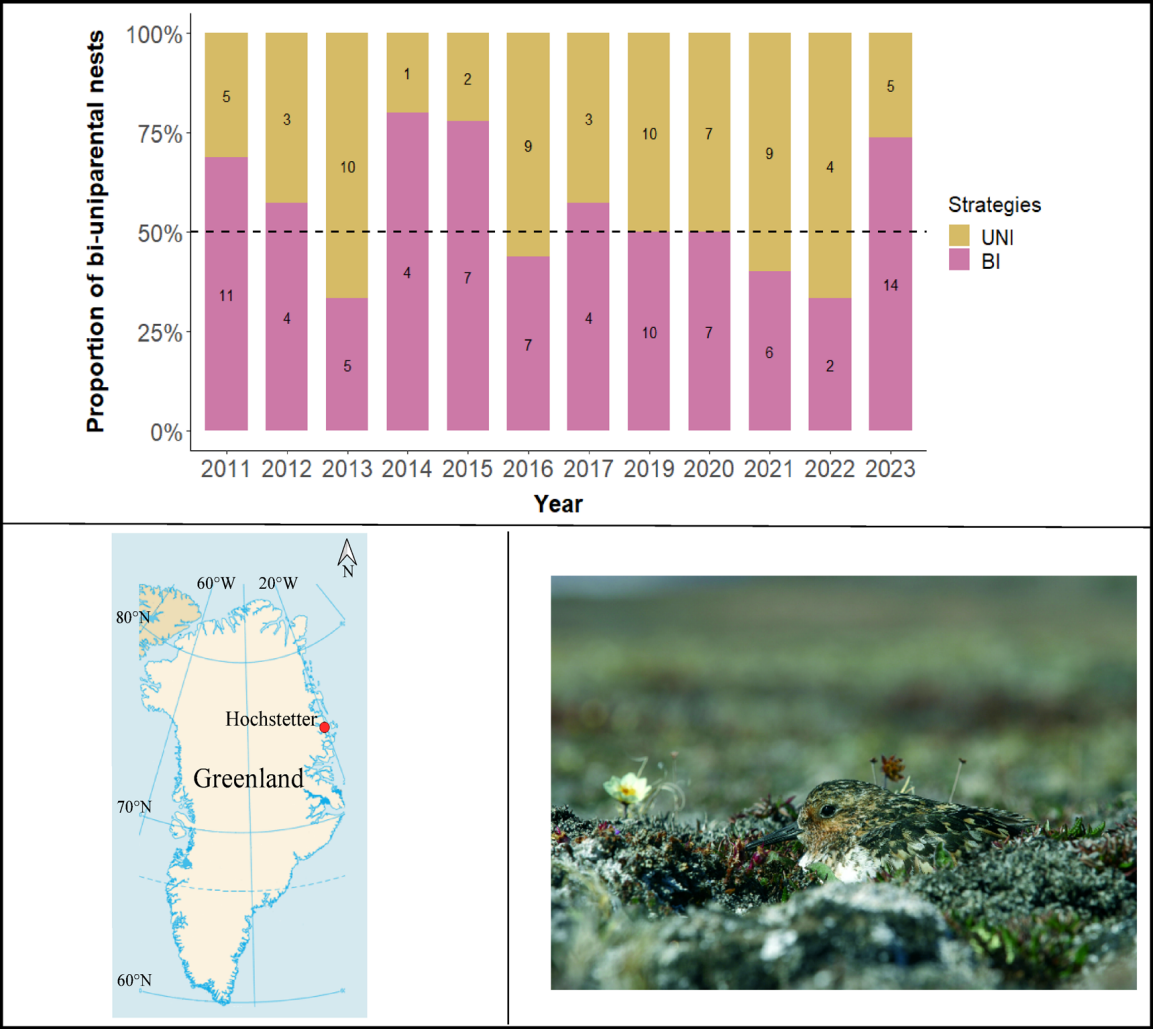
(**a**) Proportion of both incubation strategies (purple = Biparental; yellow = Uniparental) found in Sanderling nests at Hochstetter (Greenland) between 2011 and 2023 excluding 2018 (see methods; number of nests are indicated within bars). The dotted line is an indicator for the 50% of each strategy; (**b**) Location of the study area in North-East Greenland. The map was modified from https://commons.wikimedia.org/wiki/File:Greenland_blank_map.png, Creative Common licence; (**c**) Incubating sanderling (© B. Sabard and O. Gilg/GREA).

In this study, we will focus on proximal determinants of parental care strategies using Sanderlings (*Calidris alba*) during incubation, an ideal shorebird models for studying parental care decisions as both biparental and uniparental care (either males or females alone) are observed within populations at the same time^16–18^. Using a long-term dataset collected between 2011 and 2023 (Fig. 1), we aim to evaluate the direct and indirect effects of proximal variables on the inter-individual parental care variability during incubation.

**Fig. 2:**
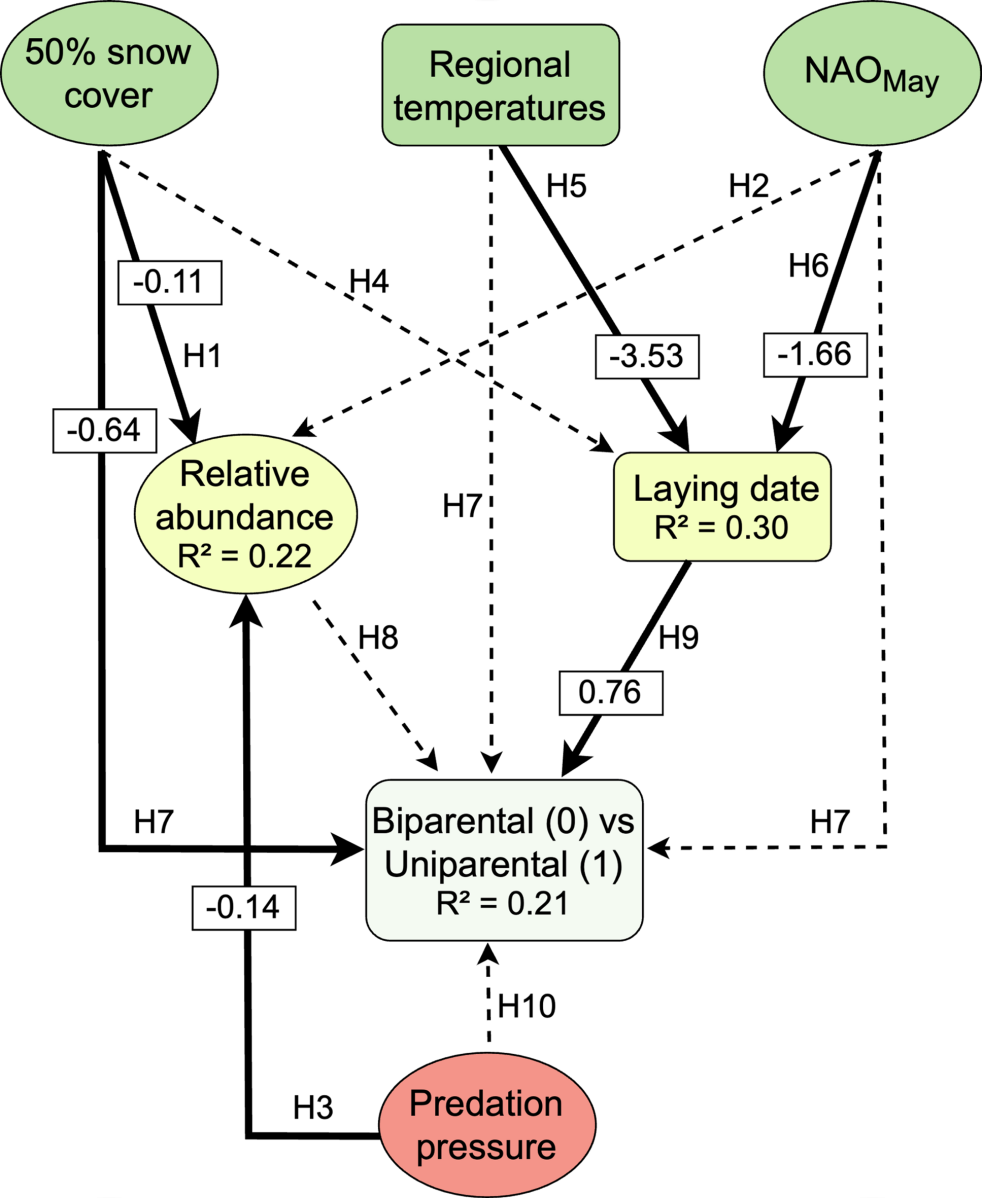
Path diagram with estimates and arrows showing the direct and indirect links between climate variables, incubation variable, predation, and incubation strategy. For the ‘Biparental vs. Uniparental’ box: Biparental = 0 and Uniparental = 1. Rounded boxes represent annual variables (i.e., one value per year) while squared ones represent individual variables (i.e., one value per nest). Bold and dotted arrows represent the direction of significant versus non-significant causal links, respectively. The corresponding hypotheses tested in our study (and detailed in Table 1) are indicated for each arrow (labelled e.g. H1, H2 in the order of Table 1). Values on the arrows show the standardised path coefficient (PC), i.e., the strength and direction of the effect. R2 represents the explained variance for each model (see Methods for details). Box colours refer to the different classes of variables (green boxes: climatic factors; yellow boxes: social environment variables; red box: predation).

We examined the effect of three categories of drivers of animal care strategies, namely ecological factors via weather, trophic interactions via predation, and social factors via partner availability (Table 1). Specifically, we tested 10 possible hypotheses about the impact of (a) ecological factors, on the relative nest abundance (H1, H2), on the laying date (measured as the laying of the first egg; H4–H6) and on parental care strategy (H7), (b) trophic interactions via predation on the availability of partner (H3) and on parental care strategy (H10), and (c) social environment on the parental care strategy (H8, H9) (see Table 1 for details). These 10 hypotheses allowed us to build a theory-grounded path diagram (Fig. 2). The connections between variables were established through a set of a priori hypotheses grounded in known mechanisms, unique to the Arctic ecosystem (see Table 1 for details on the hypotheses and their predictions).

**Table 1:** Hypothesised causal links and mechanisms between our causal and affected variables including parental care strategy, following three categories of hypotheses: ecological factor, social environment, and trophic interactions.

Category	Affected variable	Causal variable	Hypothesised mechanism and possible predictions in italics
**Ecological**	**Relative nest abundance**	**50% Snow cover**	**1. The Nesting Site-Abundance Hypothesis: Densities correlates with the availability of snow-free grounds & the phenology of snowmelt during the pre-breeding stage. The more snow-free ground available, the higher the relative nest abundance (modulates nest space available, exposure to predation & pre-breeding feeding conditions (e.g.**^19^**)**
Ecological	Relative nest abundance	NAO	2. The Macroclimate-Abundance Hypothesis: Food depletion resulting from competition at stopover sites/depleted food-supplies, and adverse weather conditions during migration can lead to substantial mortality and poor body conditions reflected in lower breeding densities^20^. NAO index in May can capture the conditions experienced by Sanderlings at the end of their migration^21^. We expect higher breeding densities when the NAO index in May is higher
**Trophic interactions**	**Relative nest abundance**	**Predation**	**3. The Predation-Abundance Hypothesis: Predators’ distribution, abundance and activity can prevent shorebirds to breed and therefore impact their distribution and abundance, both directly (via active predation) or indirectly via clues used by preys (e.g., visual or sent clues). Indeed, birds acquire information from the environment to adjust their habitat selection, behaviours, & dispersal strategies to minimise predation risk**^22,23^**. The higher the predation potential, the lower the densities**
Ecological	Laying date	50% Snow cover	4. The Nesting Site—Breeding Phenology Hypothesis: Snow-free ground are necessary to breed, hence snowmelt is a necessary condition for nest initiation (e.g.,^24,25^). We therefore expect a later snowmelt to induce delayed breeding
**Ecological**	**Laying date**	**Regional temperature**	**5. The Energetic-Breeding Phenology Hypothesis: Low temperatures during the pre-nesting period can (a) reduce the availability of arthropods to shorebirds**^26^**, and (b) increase the thermoregulatory costs for the birds**^27^**. We expect that lower temperatures would delay laying dates**
**Ecological**	**Laying date**	**NAO**	**6. The Macroclimate-Breeding Phenology Hypothesis: Weather conditions during the migration period can impact the arrival date of birds**^28,29^**, their body condition at arrival**^30^**, their clutch size**^31^**, & their overall breeding success**^31,32^**. We expect poor migration conditions (i.e., low NAO indexes) to be linked with delayed laying because of later arrival dates & altered body conditions**
**Ecological**	**Strategy**	**50% Snow cover**	**7. The Energetic Constraint Hypothesis: Under coldconditions during migration/on the breeding ground, extended snow cover during the pre-breeding period can lead to poor feeding conditions & increased energetic costs. Under these energetic constraints, we expect parental cooperation when raising the offspring, and an increasing level of biparental care (e.g.,**^6,12^**)**
Ecological	Strategy	Regional temperature	
Ecological	Strategy	NAO	
Social environment	Strategy	Relative nest abundance	8. The Re-mating Opportunities Hypothesis: Social environment shapes the mating system and parental role^6^. Availability of more potential partners may lead to an increase in re-mating opportunities, and to more uniparental nests
**Social environment /intrinsic variable**	**Strategy**	**Laying date**	**9. The Re-mating Timing Hypothesis: Having a time constrained window for reproduction, and if desertion is linked with re-mating, then earlier laying dates could allow time for other copulation attempts, increasing reproductive success**^15^**. Earlier laying dates could lead to more uniparental nests**
Trophic interactions	Strategy	Predation	10. The Predation Hypothesis: Biparental nests have a higher nest attentiveness; cryptic incubating adults might be harder to locate than unattended nests. Due to their incubation behaviour, uniparental nests have higher probabilities to be predated^33^. We would expect that under high predation pressure, more nests would be biparental

Supported hypotheses are highlighted in bold.

Here we predict that harsh environmental conditions such as cold temperatures during migration and at breeding site or late snowmelt favour biparental care^8,12,34^. Furthermore, increased predation pressure should increase parental cooperation^13^. Finally, we expect mating opportunities to influence parental care. Higher partner availability or longer breeding season should favour uniparental behaviour to increase re-mating opportunities^9,15,35^. We aimed to test these relationships and quantify their relative strengths using confirmatory path analyses^36,37^, also known as piecewise structural equation modelling^38^. To do so, we analysed 13 years of data on Sanderlings’ incubation strategy. We focused on incubation because it is when both uni-and bi-parental strategies occur. Through our analysis of 13 years of incubation data using path analysis methods, we aim to quantify the relative influence of ecological, social, and trophic factors on parental care decisions in Sanderlings, revealing how these strategies respond to the challenging conditions of the Arctic breeding environment.

## Results

### Biotic and abiotic trends

Between 2011 and 2023 (excluding 2018, see Methods), we observed an average (± SD) of 46 ± 17% uniparental nests (n = 68) and 54 ± 17% biparental nests (n = 81). We documented large interannual variation in the proportion of parental care strategies present in the breeding population, with uniparental nests ranging 20% (in 2014 & 2023) to 67% (in 2013 & 2022) and biparental nests ranging from 33% (in 2013 & 2022) to 80% (in 2014) across years (total sample size: 149 nests, Fig. 1).

Laying dates ranged from June 10th (Julian date 161; 2021) to July 18th (Julian date 200; 2012; Figure S1). Mean annual laying dates ranged from June 23rd (Julian date 174; 2021) to July 3rd (Julian date 185; 2012).

Nest relative abundance varied from 0.28 nest/km^2^ (2017) to 1.80 nest/km^2^ (2011; mean ± SD = 0.93 ± 0.45 nest/km^2^; Figure S2). The estimated daily predation rate ranged from 0 (2016) to 0.15 (2014; Figure S3).

Between 2011 and 2023 (excluding 2018, see Methods), the date of 50% snow cover ranged from June 5th (2013) to July 11th (2015; mean ± SD = 177.25 ± 11.1, Figure S4), NAO_May_ ranged from − 2.62 (2019) to 0.71 (2022; mean ± SD = -0.585 ± 1.02, Figure S5), and mean daily temperatures measured at the Daneborg weather station during the 15 days preceding laying ranged from 0 to 6.3 °C (mean ± SD = 2.20 ± 1.10; Figure S6).

### Path analyses

Following Table 1, we produced the following path diagram (Fig. 2), which was statistically supported by our data (i.e., *p* value > 0.05 for the C-statistic value; Table S2).

**Fig. 3:**
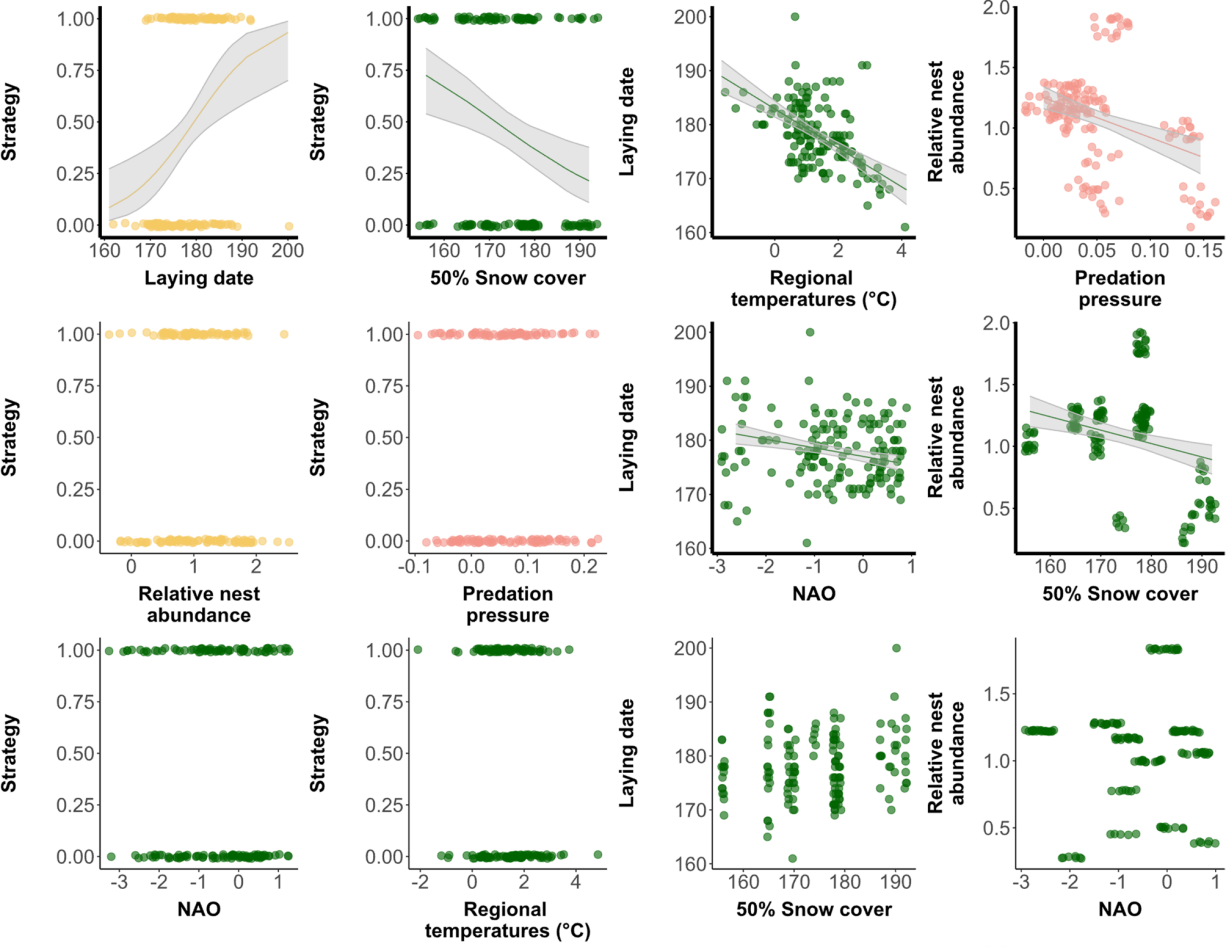
Relationships between parental care strategy (0 = Biparental, 1 = Uniparental), laying date, nest relative abundance, and their explanatory variables presented in the path diagram (Table 1; Fig. 2; n = 149 nests). Colours used in the panels refer to the different categories of hypotheses: yellow = social environment hypotheses, green = ecological factors, pink = predation. For panels presenting significant relations, the prediction of the fitted model, as modelled with the path analysis, is presented by a full line, with the shaded area representing the 95% confidence interval around the model prediction. These confidence intervals indicate the degree of certainty in the predicted relationships: narrower intervals suggest more precise predictions, while wider intervals reflect greater uncertainty. The significant positive slope in panel A shows how later laying dates increase the probability of uniparental care, while the significant negative slope in panel B demonstrates how delayed snowmelt decreases the probability of uniparental care (increases biparental care). Axes for significant relations show bold lines.

The conditions during migration, captured by the NAO index, and the regional temperature before laying, explained 30% of the variation in laying date. Regional temperatures had the largest influence on laying date, with a negative causal link two times stronger than NAO_May_ (PC_T° arctic_ = − 3.53 ± 0.46; PC_NAO_ May = − 1.66 ± 0.44; Figs. 2 and 3; supporting H5 & H6 in Table 1). We found no causal link between 50% snow cover and laying date. However, be aware that we are not working with annual mean laying dates, but individual laying dates, per nest (see Methods).

Snow cover and predation were both related to the relative abundance of nests and explained 24% of its variation (PC_predation_ = − 0.14 ± 0.03; PC_snow cover_ = − 0.11 ± 0.03; Figs. 2 & 3). Both causal links were negative, indicating that a later snowmelt and a higher predation pressure led to a lower abundance, meeting our expectations (H1 & H3 in Table 1). Although NAO_May_ was part of the causal structure, the causal link with relative abundance of nests was not significant (Figs. 2 & 3; H2 in Table 1).

Finally, parental care strategy was explained directly by both laying date (PC_laying date_ = 0.76 ± 0.24; H9 in Table 1) and snow melt (PC_snow melt_ = − 0.64 ± 0.21; Figs. 2 & 3; H7 in Table 1), and indirectly by regional temperature and NAO_May_ via laying date (products of the causal links: IE_T°-laying date-strategy_ = − 2.68; IE_NAO-laying date-strategy_ = − 1.26). Delayed laying dates resulted in more uniparental nests while delayed snow melt led to more biparental nests. Contrary to our expectations (H8 and H10 in Table 1), relative abundance of nests and predation pressure were not significantly linked to the parental care strategy. We also ran the analyses without the years with a relatively low number of nests (i.e., 2014, 2015, and 2022), and the effects on the strategies were consistent.

## Discussion

How ecological, social, and trophic factors may interact to impact inter-individual variation of parental care is unclear. Here we show that laying date and local snowmelt both influence parental care strategies, with additional indirect effects from climate conditions experienced during late migration (May) and regionally prior to egg laying. Like parental care strategies at the species-level, variations between individuals appears to be affected by ecological factors and to be constrained by the environment. However, we found no impact of our variables of social environment on parental care strategies (refuting our hypotheses # 8 & 9 in Table 1).

Being income breeders, sanderlings rely on food acquired on breeding grounds to both survive and reproduce^39^. As other long-distance migrants, they adjust their migration phenology based on conditions experienced on their wintering grounds, having no information on the local conditions they will find upon arrival on their breeding grounds^40,41^. They may hence arrive too early on a still snow-covered breeding site. Reduced food availability at arrival might result in lower body condition^42^, with negative implications for reproduction. Our path analysis revealed that delayed snowmelt led to a higher proportion of biparental nests, supporting the Energetic Constraint Hypothesis (Table 1), where harsh conditions lead to higher parental cooperation to raise the offspring (e.g.,^8,12^). Snow cover at arrival could also be used by sanderlings as a cue to anticipate later conditions and costs, rather than being an immediate energetic constraint, such as temperate birds using bud burst as a temporal clue to initiate egg laying^43^. Beyond these potential adaptive responses to snow conditions, our findings also revealed important ecological relationships at the population level. We also demonstrated that late snowmelt also negatively impacted the relative abundance of nests. This is not surprising given the very short breeding window found in high-Arctic Greenland (all but a few nests initiated within a 10-day period across the 12 years of the study; Figure S1). If Sanderlings arrive too late or if snowmelt is delayed, some individuals, or even most of them in extreme years (e.g., 2018), likely refrain from breeding^42^.

In highly seasonal environments such as the Arctic (i.e., with narrow peaks in resource availability), strong links are often found between the timing of breeding in insectivorous birds and the availability of arthropods (e.g.,^44–46^), the phenology and abundance of the latter being dependent on local temperatures^27^. Our results are in line with these findings (see negative relation between regional temperature and laying date in Fig. 2). Furthermore, the timing of breeding is also strongly constrained by the short summers and by changing migration phenology^47^. Indeed, inclement weather conditions during the final legs of the migration journey can delay arrival times^28,29^ and explain later laying dates^48^, as supported by our results on Sanderlings (see negative relation between NAO_May_ and laying date in Fig. 2). Considering all these constraints, delayed laying dates are potentially very harmful for the fitness of migrating arctic shorebirds (i.e., for their own survival and their breeding success), especially in the current context of climate change which increases the risk of trophic mismatch^46,49^, but see^50,51^. Still, laying later can also present some advantages, e.g. regarding nest site selection (i.e., more snow free areas available) and predation (i.e., dilution effect after snowmelt versus high predation pressure on the first snow free patches during the onset of snowmelt^52^). Furthermore, laying later may offer milder temperatures, which is advantageous for the energetic balance of both the parents and the eggs.

By sharing parental responsibilities early in the season, biparental pairs could be better poised to navigate the challenges posed by unfavorable environmental and energetic conditions. Conversely, most uniparental birds are probably only able to cover all incubation costs on their own once these constraints are partially relaxed, and therefore costs are lower, i.e., later in the season.

The timing of breeding can also be investigated in the light of re-mating opportunities^15,35,53,54^: (1) higher abundance is assumed to offer more re-mating opportunities and (2) earlier laying dates could allow successive breeding attempts during the same breeding season^15^, both situations leading to a higher proportion of uniparental nests at the onset of the laying period. Our results do not support these hypotheses (i.e. the lack of relation between Relative abundance and incubation strategy, and the positive relation between Laying date and the proportion of uniparental nests; Fig. 2). The short breeding season typical of the Arctic environment, exacerbated by migratory constraints and harsh local conditions, therefore seems to be the key to explain the incubation strategies of Sanderlings.

### Future directions

This study lays the groundwork for additional research on parental care strategies in Arctic shorebirds. Future work should focus on collecting detailed data on individual body condition, and fine-scale arthropod availability to further test the energy constraint hypothesis and understand the role of body condition in the decision-making process. Insights into foraging behavior, territory sizes, and home ranges would enhance our understanding of resource acquisition patterns and their impact on parental care decisions. Additionally, comprehensive population-level data including non-breeding individuals would allow examination of how density and sex ratios influence breeding strategies. A comparative approach across a latitudinal gradient could reveal how reproductive windows affect parental care strategies under varying environmental conditions, thus shedding light on individual decision-making processes.

## Conclusion

Our study investigates parental care flexibility in Sanderlings, a species where uniparental and biparental strategies coexist with changing proportions across years. In our study site, we demonstrated that these proportional shifts are primarily shaped by ecological factors and energetic constraints rather than by social factors or predation pressure. Overall, harsh arctic environmental conditions seem to favour the biparental (i.e., cooperative) strategy between parents. Other classically invoked drivers, such as predation or relative abundance, have no or only limited effect in our system. Overall, these findings underscore the complex interplay between environmental factors and parental care strategies in shorebirds, offering insights into how these strategies are likely to respond to rapidly changing Arctic ecological conditions driven by climate change.

## Materials and methods

### Study site

The study was conducted over a 13-year period (2011–2023) at Hochstetter Forland, Northeast Greenland (75.15°N 19.70°W) on a 18 km^2^ study area. Winters in northeast Greenland are characterised by very cold temperatures, ranging between − 25 and − 15 °C. However, during the Sanderling’s breeding season, temperatures rise above 0 °C, with average monthly temperatures between 2 and 4 °C^55^. The study site is within the Northeast Greenland National Park, an area with minimal human impact.

### Sanderling monitoring

#### Incubation strategy

The Sanderling is a small (44–71 g) long-distance migratory shorebird breeding in the High Arctic^56^ (Figure S3c). This species exhibits a mixed incubation strategy, with both biparental and uniparental care observed, and with both sexes able to incubate and rear chicks^16,17,57,58^. To provide context for our study of incubation strategies, we first outline key aspects of Sanderling breeding phenology. While there is little data on the exact breeding phenology of Sanderlings^59^, they are known to arrive on their Arctic breeding grounds pending favourable snow thaw, typically from late May to early June. For income breeders like the Sanderling, access to snow-free areas is critical as they rely upon local resources to meet the energy demand of egg production (e.g.^60^). Pairs form quickly, and egg laying begins within a week to two weeks after arriving from migration^61^. Incubation of the 4 eggs (sometimes 3) lasts approximately 21.5 days (this study and^62^). Post-hatching, the limited available data suggest up to three weeks of chick rearing^61,63^.

In all years, nests were searched during the incubation period (late June to late July), in the same study area (18 km^2^). They were searched in suitable habitats and located opportunistically by flushing incubating adults or by following birds with anti-predator behaviour^64^. In each nest, we monitored incubation behaviour using a temperature probe (Flylead thermistor PB 5009 with 60 cm cable) coupled to a data logger (Tinytag Plus2 TGP-4020; Gemini Data Loggers Inc., West Sussex, U.K.; see full methods^18^). In 2018, a year characterised by very late snowmelt and very poor breeding conditions (see^42^), we only found two late nests; therefore 2018 was excluded for the analyses. A total of 149 nests was discovered and used for this study (see Fig. 1 for annual distribution). Annual permits for Sanderling’s research were granted by the Government of Greenland, Ministry of Domestic Affairs, Nature and Environment-NNPAN, for Hochstetter (permit numbers: C-11-4-12, C-12-4-17, C-13-4-29, C-14-4-23, C-15-4-10, C-16-4-15, C-17-3-28, C-18-3-11, C-19-3-03, C-20-3-19, C-21-3-22).

Uniparental and biparental incubation strategies were assigned to each nest, following^65^. This approach uses a discriminant equation that considers the daily number and duration of recesses observed in the nests, uniparental birds leaving the nest longer and more often than biparental ones. Out of the 149 nests used in this study, we also used 23 nests that started as biparental but where one partner deserted during the incubation period. This is a common behaviour in species using both incubation strategies^66^. Although their status eventually changed to uniparental, we classified them as biparental in our analyses, as they remained so within the timeframe used for path analysis. We report this later change for transparency; however, it had no bearing on our results, as the desertion occurred outside the analytical window.

#### Laying date

As most other Arctic sandpipers do, Sanderlings usually lay four eggs (sometimes only three^67^), lay one egg per day, and start incubating after having laid the penultimate egg^68^. We used three complementary methods to estimate laying date (i.e., laying date of the very first egg). For nests discovered during laying, we assumed a laying rate of one egg per day to determine the initiation of laying. For nests discovered before the clutch was completed (total clutch size confirmed during subsequent visits), we simply subtracted one day from the observation date for every egg found in the nest. For other nests, where hatching was documented with the thermoprobes (see above), we subtracted the average duration of incubation for the species (i.e., 21.5 days^69^), plus two or three days (for nests with three or four eggs, respectively), from the hatching date. Finally, if none of the previous methods could be applied, we used egg flotation^70,71^ to estimate the day when incubation began (assuming again an average incubation period of 21.5 days), and subtracted an additional two or three days as above (see Supplementary in^17^ for more details). Egg flotation is known not to impact hatching success^72^.

### Relative nest abundance

Every year, nests were searched with similar searching effort (six persons in all years but 2015 with 2 persons), over the same study area (18 km^2^), with the same protocol^73^. Searching over such a large study area is mandatory to reach good sample sizes for a species whose breeding densities are very low in the high-Arctic tundra. Since some incubating birds do not flush until you are closer than one metre from their nest, it cannot be used to assess true nest densities. Results of our annual surveys are hence only considered as relative nest abundances.

### Predation pressure

We used the inverse of the mean daily survival rate (DSR), estimated for each year, with the classical Mayfield method^74,75^ implemented in the program MARK^76^, as a proxy for predation pressure (i.e., 1—DSR; Table S1). DSR is calculated as the total number of predated nests during a breeding season divided by the total number of exposure days^77,78^. For each study year, DSR hence gives us the mean daily survival rate of a nest estimated from the entire breeding population, while taking the inverse (1—DSR) gives us the mean daily predation rate of a nest estimated for the same year and population. The main predator of shorebirds eggs in northeast Greenland is the Arctic fox (*Vulpes lagopus*), although several species of avian predators are also known to feed on shorebird eggs (Long-tailed skuas, *Stercorarius longicaudus*; Arctic skuas, *Stercorarius parasiticus*; gulls, *Larus spp;* Raven, *Corvus corax*)^79,80^.

### Climate data

#### Weather conditions during spring migration

We used the North Atlantic Oscillation (NAO) index^81^ in May to encapsulate the overall weather conditions encountered by Sanderlings during the final part of their spring migration^21^ (see Table S1). The NAO index reflects the large-scale fluctuations in atmospheric masses over the North Atlantic, by comparing atmospheric pressures measured in Iceland and in the Azores. It is considered as the most significant atmospheric oscillation in the North Atlantic region^82^. NAO index can be either positive or negative, with positive values reflecting warm and wet conditions, while negative values reflect colder and drier conditions.

#### Regional temperatures

Temperature data were retrieved from the Danish Meteorological Institute data repository^83^ (Table S1). We used the data from the nearest weather station, Daneborg (74.31° N, 20.22° W), located 95 km south of our study area. To test the homogeneity of the climate at the regional scale and hence confirm that Daneborg could be reliably used to reflect changes in daily temperatures at our site, we compared Daneborg temperatures with temperatures measured at the second nearest weather station, Danmarkshavn (76.77° N, 18.68° W), located 185 km north of our study area. Both time series were highly correlated (R^2^ = 0.94, *p* < 0.001). We first extracted daily mean air temperatures recorded every hour at 20 m above sea level. For each monitored nest, we then computed the mean daily temperature during the 15 days preceding the laying date to represent the pre-breeding conditions faced by sanderlings.

#### Snow cover

For each year, we extracted the Julian date of 50% of snow cover using MODIS satellite data (Table S1) via the ‘MODIStsp’ package^84^ in R^85^. This product is based on the MOD10A1, Terra Snow Cover Daily L3 Global 500 m SIN Grid, Version 6.1^86^. It offers a daily composite of snow cover derived from the ‘MODIS/Terra Snow Cover 5-Min L2 Swath 500 m’ data set (DOI: 10.5067/MODIS/MOD10_L2.061). Each data granule is a 10° × 10° tile projected to a 500 m sinusoidal grid. We used the smallest tile possible including our study area. We collected data from the beginning of June until mid-August and determined the date when 50% of the study area was snow free. Using the R package ‘raster’^87^, we detected and removed cloud and ocean pixels from the data. Snow cover for days with overcast sky was estimated from linear interpolation between the last and next images available for cloud free days. As documented by Gauthier et al*.*^88^, this approach provides reliable estimates of local spring snow cover. Snow cover, unlike temperature, is not extracted for each nest since it is a landscape-scale metric and our analyses contain only one annual value for all tracked nests.

### Statistical analyses

We used confirmatory path analyses to test our causal hypotheses presented in Table 1^36^. This approach allows us to represent all causal linkages between variables, with both direct and indirect effects, in one unified network. Unlike standard regression methods, path analysis allows variables to serve as both predictors and responses within the same analytical framework. This distinction is critical for our study because factors like snow cover can directly influence parental care strategies while also affecting other variables such as laying date, which in turn affects parental care.

We defined our causal structure based on established ecological theory and prior knowledge of Arctic breeding systems. Each hypothesized pathway (Fig. 2) represents a specific ecological relationship outlined in Table 1. Direct effects occur when one variable influences another without intermediaries, while indirect effects operate through mediating variables. This approach allowed us to quantify the relative strength of each pathway and identify which environmental factors have the strongest influence on parental care decisions.

We tested associations between different variables related to climate, predation, and incubation behaviour of Sanderlings between 2011 and 2023 (excluding 2018), based on our a priori knowledge of the system, and the availability of the variables, as previously done (e.g.,^37^). We defined our causal hypotheses and specified hypothesised mechanisms and predictions in Table 1 and expressed these hypotheses in a directed acyclic path diagram (Figs. 1 & 2). We then translated the path diagram into path models, composed of a set of statistical models, i.e., one for each variable having at least one causal parent: relative nest abundance, laying date, and incubation strategy. We used the package PiecewiseSEM^38^ in R (version 4.3.0^85^) to translate the diagram in models and this simplifies into three models that run in the main function of the package (psem): First, we have a linear model for relative nest abundance as a response variable, and NAO in May, predation , and snow cover as predictors. Second, we have a linear model for laying date as a response variable, and NAO in May, local temperature, and snow cover as predictors. For those two linear models, we used the adjusted-R^2^ in Fig. 2, which accounts for the comparison of models with different numbers of predictors. Third, we also use a generalised model for incubation strategy as a response variable (as a binomial distribution, coded as biparental being 0 and uniparental being 1), with NAO in May, local temperature, the relative nest abundance, predation, laying date, and snow cover as predictors, Since this model is logistic, the psem function of PiecewiseSEM provides an approximation of the R^2^, i.e. the Nagelkerke R^2^. We built our models by avoiding multicollinearity following the criteria of Zuur et al.^89^. In the results, we present the standardized path coefficient (coefficient’s estimate adjusted by its standard deviation), which allows direct comparison of the relative magnitude of the effects of the different explanatory variables in the pathway.

## Electronic supplementary material

Below is the link to the electronic supplementary material.

Supplementary Material 1

## Data Availability

The data and R code are publicly archived on Dryad (https://datadryad.org/stash/share/bPcakUHiG60ACAH9UDAXujIoAohzd5jnRTYw-hIPXHQ), DOI: 10.5061/dryad.v6wwpzh4
