# Supplementary material for: Snowmelt and laying date impact the parental care strategy of a high-Arctic shorebird
Source: Sci Rep. 2025 Jun 5;15:19760. doi: 10.1038/s41598-025-02318-y (PMC12141443; doi:10.1038/s41598-025-02318-y)
Supplement: Supplementary file 1 — Supplementary Material 1 [file 41598_2025_2318_MOESM1_ESM.docx]

**Snowmelt and laying date impact the parental care strategy of a high-Arctic shorebird**

**Léa Etchart^1,2^, Nicolas Lecomte*^2^, François-Xavier Dechaume-Moncharmont^3^, Johannes Lang^4,5^, Jérôme Moreau^5,6,7^, Thomas Pagnon^1,5^, Niels Martin Schmidt^8^, Benoit Sittler^5,9^, Loïc Bollache^1,5^, Olivier Gilg^1,5^**

^1^**Université Marie et Louis Pasteur, CNRS, Chrono-environnement (UMR 6249), F-25000 Besançon, France**

^2^Canada Research Chair in Polar and Boreal Ecology and Centre d'Études Nordiques, Université de Moncton, Moncton, NB, Canada
^3^Universite Claude Bernard Lyon 1, CNRS, ENTPE, UMR 5023, Villeurbanne, France
^4^Working Group for Wildlife Research at the Clinic for Birds, Reptiles, Amphibians and Fish, Justus Liebig, University Giessen, 35392 Giessen, Germany
^5^Groupe de Recherche en Écologie Arctique, 21440 Francheville, France
^6^UMR 6282 CNRS, Université de Bourgogne, 6 boulevard Gabriel, Dijon, France
^7^Centre d’Études Biologiques de Chizé, CNRS, 79360 Villiers en Bois, France
^8^Department of Ecoscience and Arctic Research Centre, Aarhus University, Roskilde, Denmark
^9^Chair for Nature Conservation and Landscape Ecology, University of Freiburg, Freiburg, Germany

Léa Etchart: etchart.lea@gmail.com. ORCID: 0009-0004-0095-1196
Nicolas Lecomte: nicolas.lecomte@umoncton.ca. ORCID: 0000-0002-8473-5375
François-Xavier Dechaume-Moncharmont: fx.dechaume@univ-lyon1.fr. ORCID: 0000-0001-7607-8224
Johannes Lang: johannes.lang@vetmed.uni-giessen.de. ORCID: 0000-0002-7387-795X
Jérôme Moreau: jerome.moreau@u-bourgogne.fr. ORCID: 0000-0001-9984-0998
Thomas Pagnon: thomas.pagnon@gmail.com. ORCID: 0009-0003-1204-5230
Niels Martin Schmidt: nms@ecos.au.dk. ORCID: 0000-0002-4166-6218
Benoit Sittler: benoit.sittler@nature.uni-freiburg.de.
Loïc Bollache: loic.bollache@u-bourgogne.fr. ORCID: 0000-0003-0316-6746
Olivier Gilg: olivier.gilg@gmail.com. ORCID: 0000-0002-9083-4492

*Corresponding author at: Canada Research Chair in Polar and Boreal Ecology and Centre d'Études Nordiques, Université de Moncton, Moncton, NB, Canada

Email address: [Nicolas.Lecomte@umoncton.ca](mailto:Nicolas.Lecomte@umoncton.ca) (N. Lecomte)

# Supplementary information

This document includes:

- Supplementary Tables
- Supplementary Figure

# Table S1. Description of the variables used in path analyses and source of the data.

| **Type** | **Variable** | **Name** | **Sampling** | **Details** | **Origin** |
| --- | --- | --- | --- | --- | --- |
| Migration conditions | NAO_May_ | *nao_may* | Seasonal | Global climatic conditions during the peak migration month (May) of Sanderlings (Reneerkens et al., 2009) | NOAA* |
| Predation pressure | 1 – DSR (Daily survival rate) | *predation* |  | DSR = total number of predated nests during a breeding season / total number of exposure days (Rotella, 2019). | Field monitoring |
| Regional climate | 50% Snow cover | *snow_cover* | Daily | Julian date of 50% of snow melt per year | MODIS** |
|  | Local temperatures | *local_temperature* |  | Mean of the residuals of the relationship Daily temperature ~ Julian date of each monitored nest the 15-days prior to the laying | DMI*** |
| Nest variables | Relative nest abundance | *relative_abundane* | Incubation | Total number of nest found in the study area (/km²) | Field monitoring |
|  | Laying date | *laying_date* |  | Field observations and back calculator according to four methods, see Methods |  |
|  | Incubation strategy | *dummy_strat* |  | Determined incubation strategy (uniparental or biparental) based on TinyTag records (Moreau et al., 2018) |  |

* <https://www.cpc.ncep.noaa.gov/products/precip/CWlink/pna/norm.nao.monthly.b5001.current.ascii.table>

** https://nsidc.org/data/mod10a1f/versions/61#anchor-1

*** <https://confluence.govcloud.dk/display/FDAPI/Climate+Data>

Table S2. Summary of causal links’ estimates of our path analysis shown in Table 1 and Figure 3. Standardised path Coefficient (std PC) for each causal link with their standard error (SE) are shown. Significant path coefficients are highlighted in bold.

| Response variable | Predictor | | std PC | SE | p-value |
| --- | --- | --- | --- | --- | --- |
| Strategy  Laying date  Nest density | | NAO | -0.05 | 0.21 | 0.8 |
|  |  | Temperature (°C) | -0.03 | 0.27 | 0.9 |
|  |  | Nest density | -0.16 | 0.21 | 0.5 |
|  |  | Predation | -0.21 | 0.21 | 0.3 |
|  |  | **Laying date** | **0.76** | **0.24** | **< 0.005** |
|  |  | **50% snow melt** | **-0.64** | **0.21** | **< 0.005** |
|  |  | **Temperature (°C)** | **-3.53** | **0.46** | **< 0.001** |
|  |  | **NAO** | **-1.66** | **0.44** | **< 0.001** |
|  |  | 50% snow melt | -0.01 | 0.43 | 1.0 |
|  |  | NAO | -0.002 | 0.03 | 0.9 |
|  |  | **Predation** | **-0.14** | **0.03** | **< 0.001** |
|  |  | **50% snow melt** | **-0.11** | **0.03** | **< 0.001** |


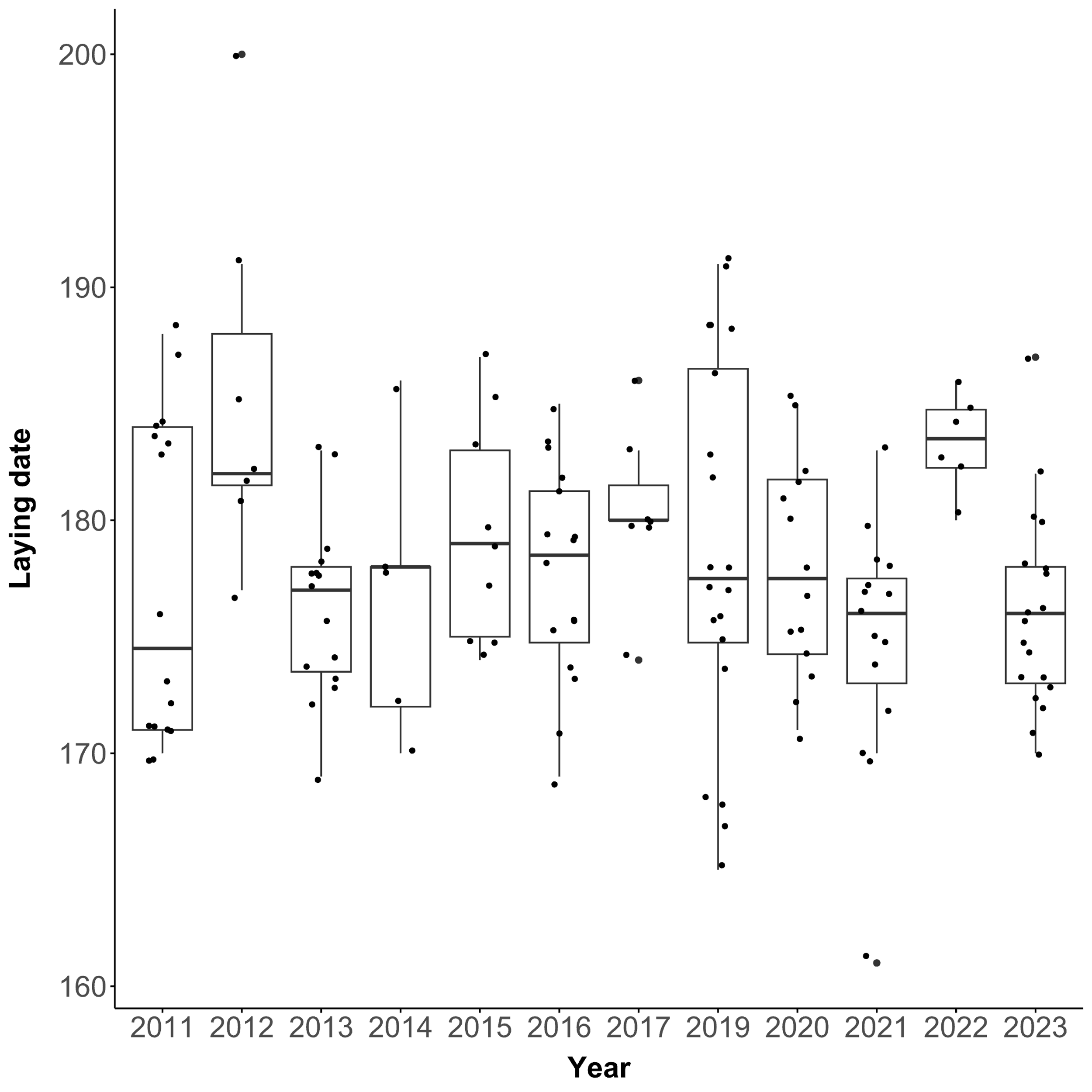


Figure S1. Distribution of laying date per year between 2011 and 2023. Julian date 160 = June 9^th^, 170 = June 19^th^, 180 = June 29^th^, 190 = July 9^th^, 200 = July 19^th^.


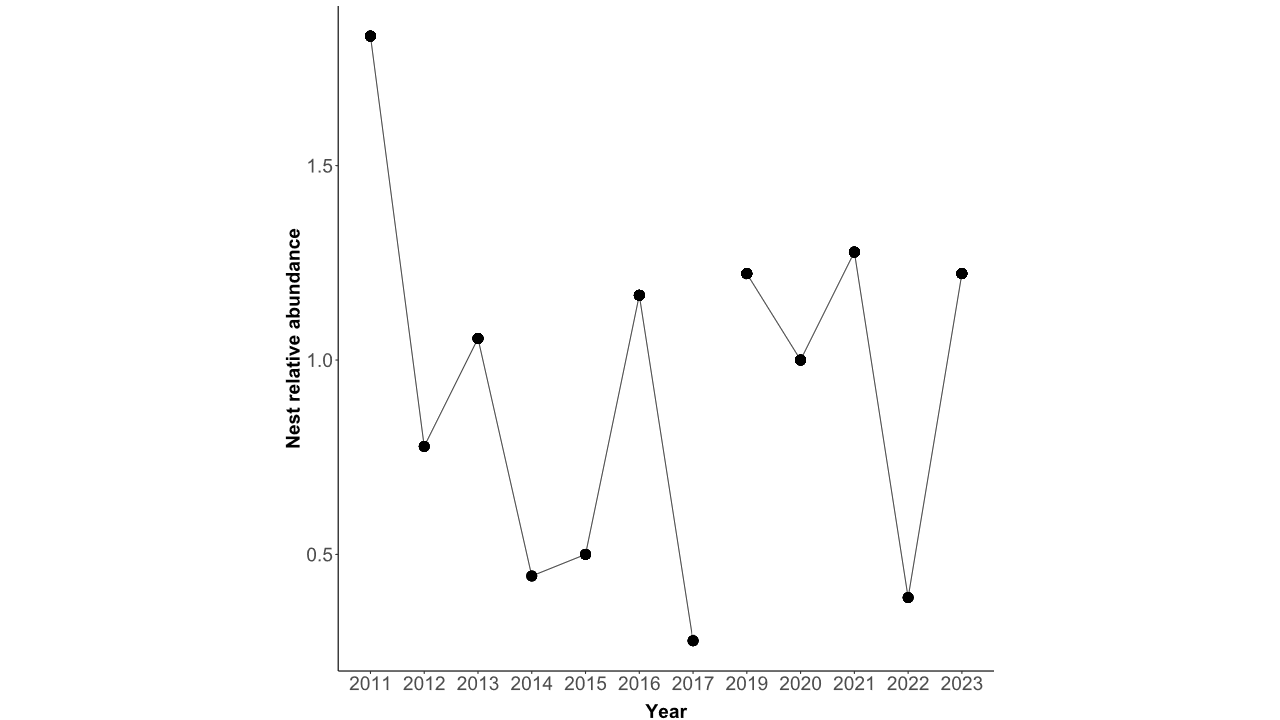
Figure S2. Annual nest density of Sanderlings (number of nests /km²), in Hochstetter (Greenland) between 2011 and 2023.


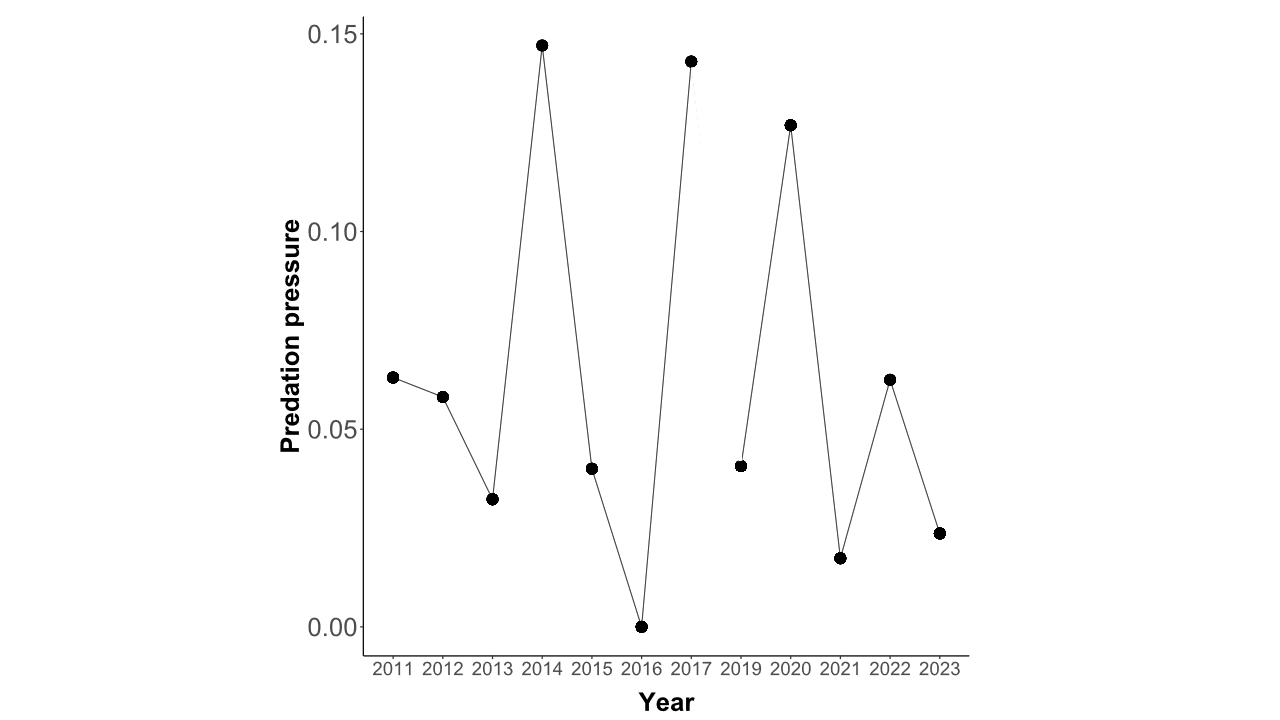
Figure S3. Annual predation pressure (1 – DSR, see Methods) of both terrestrial (Arctic fox) and avian predators (Gulls, corvids, and skuas) on Sanderling nests in Hochstetter (Greenland) between 2011 and 2023.


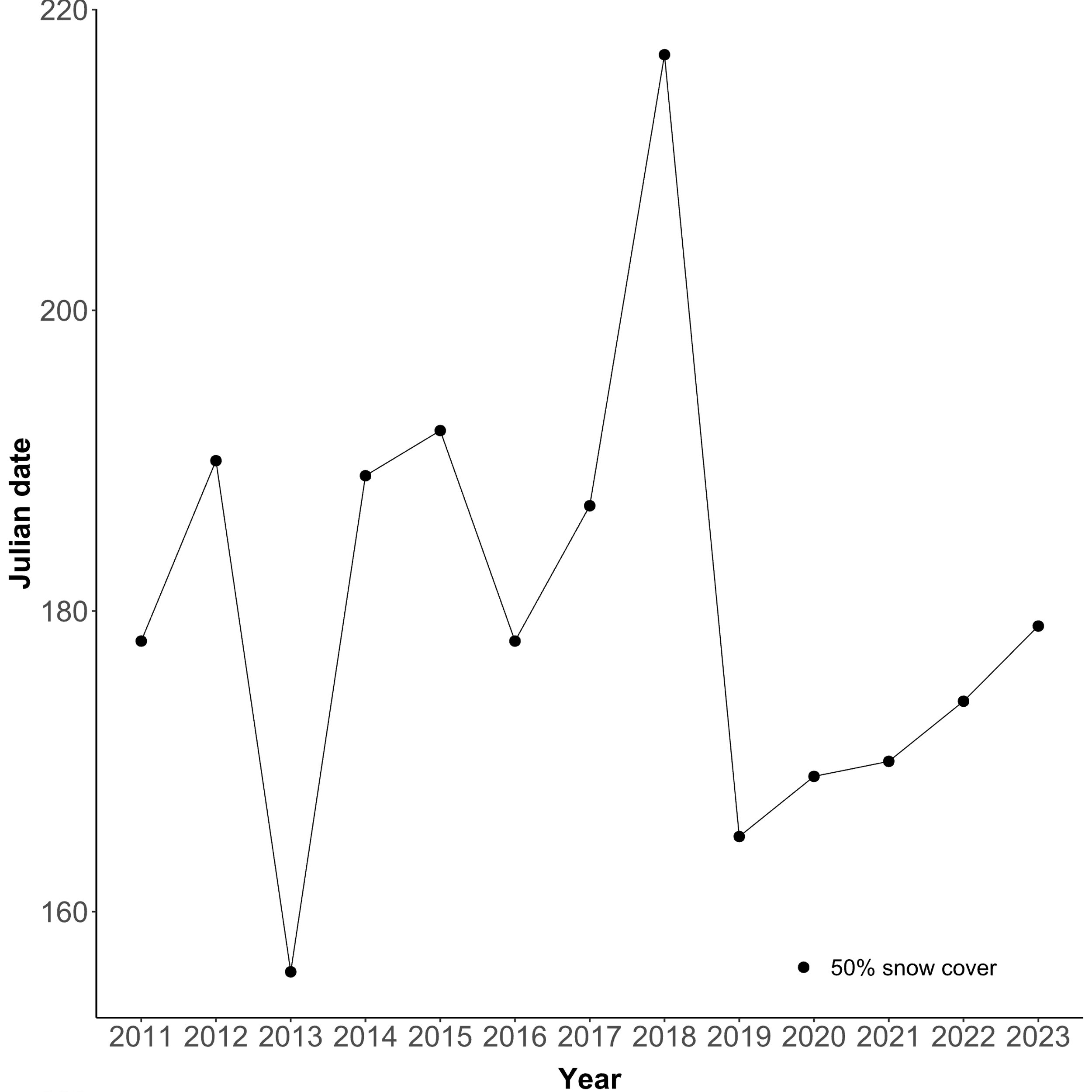
Figure S4. Annual values of 50% snow cover in Hochstetter (Greenland) between 2011 and 2023. Julian date 160 = June 9^th^, 180 = June 29^th^, 200 = July 19^th^, 220 = August 8^th^.


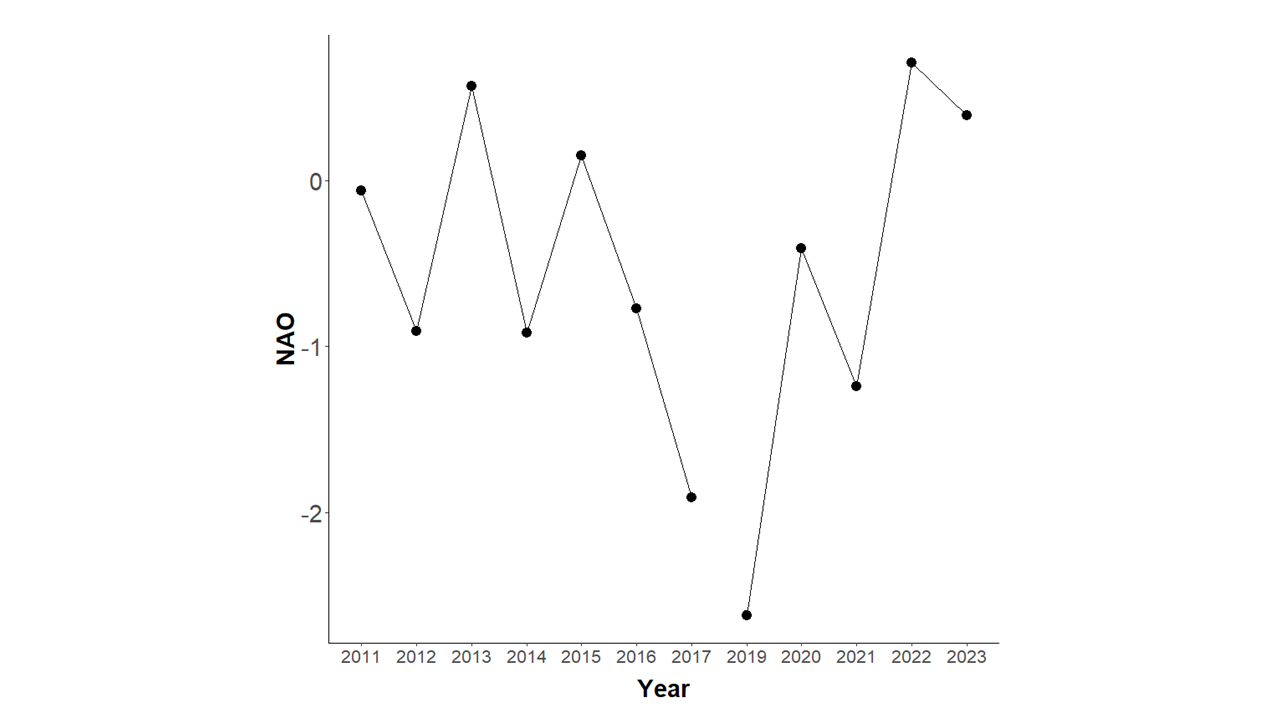
Figure S5. NAO_May_ values for each monitored year (2011-2023) to represent migration condition of Sanderlings coming from wintering areas, mostly flying through Iceland.


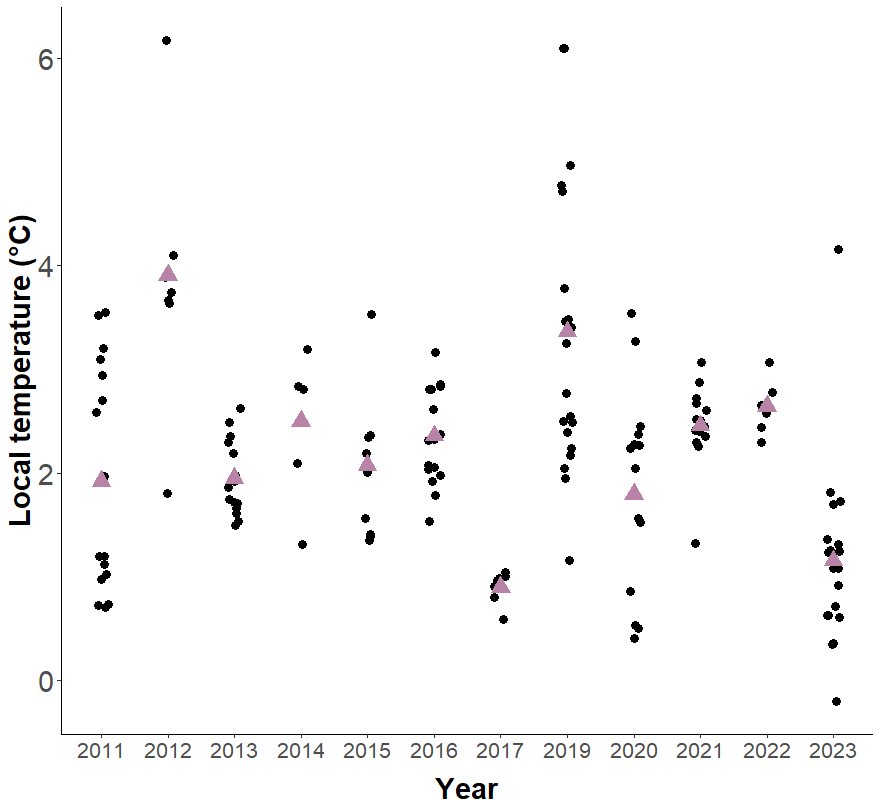


Figure S6. Mean temperatures of the 15-days preceding the laying for each monitored nest of Sanderlings per year in Hochstetter (Greenland) between 2011 and 2023. Black dots represent each nest’s temperature and pink triangles represent the mean temperature faced by all nests by year.
